# Caution is required when using health facility-based data to evaluate the health impact of malaria control efforts in Africa

**DOI:** 10.1186/1475-2875-8-209

**Published:** 2009-09-03

**Authors:** Alexander K Rowe, S Patrick Kachur, Steven S Yoon, Matthew Lynch, Laurence Slutsker, Richard W Steketee

**Affiliations:** 1 Malaria Branch, Division of Parasitic Diseases, National Center for Infectious Diseases, Centers for Disease Control and Prevention (CDC), Atlanta, Georgia, USA; 2 Global Program on Malaria, Center for Communication Programs, Bloomberg School of Public Health, Johns Hopkins University, Baltimore, Maryland, USA; 3 Program for Appropriate Technology in Health (PATH), Ferney-Voltaire, France

## Abstract

The global health community is interested in the health impact of the billions of dollars invested to fight malaria in Africa. A recent publication used trends in malaria cases and deaths based on health facility records to evaluate the impact of malaria control efforts in Rwanda and Ethiopia. Although the authors demonstrate the use of facility-based data to estimate the impact of malaria control efforts, they also illustrate several pitfalls of such analyses that should be avoided, minimized, or actively acknowledged. A critique of this analysis is presented because many country programmes and donors are interested in evaluating programmatic impact with facility-based data. Key concerns related to: 1) clarifying the objective of the analysis; 2) data validity; 3) data representativeness; 4) the exploration of trends in factors that could influence malaria rates and thus confound the relationship between intervention scale-up and the observed changes in malaria outcomes; 5) the analytic approaches, including small numbers of patient outcomes, selective reporting of results, and choice of statistical and modeling methods; and 6) internal inconsistency on the strength and interpretation of the data. In conclusion, evaluations of malaria burden reduction using facility-based data could be very helpful, but those data should be collected, analysed, and interpreted with care, transparency, and a full recognition of their limitations.

## Background

Otten and colleagues [1] recently published an evaluation of the impact of malaria control in Rwanda and Ethiopia. This article is timely, as the global health community has considerable interest in the health impact of the billions of dollars invested to fight malaria in Africa. To date, few evaluations have been published, mostly of malaria programmes in relatively small islands, such as Bioko [2] and Zanzibar [3]. Otten and colleagues present trends in malaria cases and deaths from health facility records as evidence that the scale-up of long-lasting insecticidal nets (LLINs) and case management with artemisinin-based combination therapy (ACT) reduced the burden of malaria. Although the authors demonstrate the use of facility-based data to estimate the impact of malaria control efforts, they also illustrate several pitfalls of such analyses that should be avoided, minimized, or actively acknowledged. While news of malaria control success is appreciated, in this Commentary, a critique of this analysis is presented because many country programmes and donors are interested in evaluating programmatic impact with facility-based data, which are available in most countries in sub-Saharan Africa.

## Discussion

The ideal method for assessing impact requires that observed changes in malaria morbidity and mortality (based on perfectly valid data) are attributed to exposure to an intervention(s). An experimental study design is needed to assess what would have happened had that exposure never occurred. Although it is highly unlikely that evaluations of real-world programmes would involve such methods, an examination of how an evaluation deviates from the ideal can be used to judge the evaluation's validity. Note that it should be mentioned that programme evaluations can still be robust even if ideal experimental designs are not used.

The first concern of the evaluation by Otten and colleagues is that it is unclear whether the objective was to assess the programmatic impact on the malaria burden at health facilities or in communities. The background states that the authors intended to assess the impact of malaria control on "health facility burdens"; however the article does not explain what this means, and the implication is that the facility-based results reflect malaria trends in the community. Although the terms "health facility burden" and "community burden" might not have formally recognized definitions, the former typically refers to caseloads (e.g., cases of malaria and anaemia), commodity use, and costs incurred in health facilities; and the latter means malaria cases and deaths in the general population. While both types of burden are important, a trend in one does not necessarily imply a corresponding trend in the other. For example, a community case-management programme that primarily shifts care-seeking from facilities to village-level providers could decrease the health facility burden with little effect on the community burden. More importantly, while accurate and complete health facility records are an excellent data source for evaluating changes in the health facility burden, these data might not produce valid trends for the community burden.

The second concern is that a lack of detail in the article makes it difficult to judge the validity of the data. For example, in Rwanda, the statement that "all sampled facilities performed malaria smears on all suspected malaria cases" does not seem plausible. Not a single patient was missed--even during weekends and evenings? Nationally, according to Rwanda's Health Management Information System, in 2007, only 45% of facility-based malaria cases were laboratory-confirmed [4]. Also, in 2006, Rwanda adopted the World Health Organization's Integrated Management of Childhood Illness strategy [5], which does not recommend routine malaria testing for children with a febrile illness who are under five years of age. With such a strategy, most children would not be tested. Accounting for testing trends is critical because increased testing can dramatically decrease the incidence of malaria diagnoses (i.e., malaria is increasingly ruled out among patients with febrile illness that might have been previously reported as malaria cases), and both countries recently made efforts to increase malaria testing. Additionally, the article did not describe the quality of diagnostic testing (e.g., sensitivity and specificity) and whether quality changed over time or differed from place to place. Changes in testing quality could bias trends in malaria outcomes (e.g., microscopy training that decreased false-positives would lead to declines in observed cases even if the true rate was unchanged). It would have been helpful if the authors had described how they determined that all suspected cases were tested and characterized the use and quality of diagnostic testing over time. Data validity was even more difficult to assess for Ethiopia, as laboratory examinations were not recorded among outpatients and the availability of laboratory data for inpatients was not mentioned.

Third, the sampling procedures make it difficult to assess the representativeness of the data. The authors stratified their convenience sample so that selected facilities would be spread out across malarious areas of both countries. However, the selection of "sites where intervention scale-up had been relatively rapid and successful and where health facility data were of relatively good quality" suggests that results were biased toward areas likely to have a relatively greater impact. Additionally, the number of health facilities was small: 19 in Rwanda and 13 in Ethiopia. With such small samples, even the use of probability sampling does not guard against skewed results.

Fourth, the analysis did not include trends in factors that could influence malaria rates and thus confound the relationship between LLIN and ACT scale-up and the observed changes in malaria outcomes. Key examples of such factors are rainfall, implementation of a home-based fever treatment strategy, and indoor residual spraying--all of which could have changed the rate of malaria cases seen at health facilities, but not necessarily cases of other illnesses. Even a simple graph of such trends over time together with the malaria outcomes can be helpful in understanding the potential effect of these factors (see example in Bhattarai and colleagues [3]).

Fifth, some of the analytic approaches raised concerns. For example, at least one conclusion was based on a very small number of patient outcomes. In Ethiopia, for children under five years of age, the reported impact on inpatient deaths was based on a decrease of 11 deaths per year during the reference period to four deaths in 2007. Another issue with the analytic approach was that there appeared to be some selective reporting of results, in which decreases in malaria (e.g., from 2005-2007 in Figure four, and from 2006-2007 in Figure five) were attributed to the scale-up of LLINs and ACT, but similar decreases (e.g., from 2001-2002 in Figure four, and from early 2001-2003 in Figure five) were not discussed.

Additionally, the statistical methods were not ideal and might have led to an underestimation of uncertainty. The methods did not account for the correlated nature of the data (i.e., the data were repeated measures of the malaria caseload at selected health facilities over time), and a failure to adjust for correlation (e.g., with generalized estimating equations or a random-effects model) could make results appear more precise than they actually are. Another statistical issue was the use of linear regression to model counts when a non-linear model (e.g., based on a Poisson or negative binomial distribution) would have been more appropriate. For example, if the trend for inpatient cases in Figure four continued for another year or two, a linear model probably would have predicted a negative case count (obviously impossible). Perhaps, if more suitable methods had been used, the decline of outpatient malaria cases in Ethiopia, adjusted for linear trend (69%; 95% confidence interval: 45-83%), would no longer be statistically significant. Indeed, to us, the 2007 data point of outpatient malaria cases in Figure four appeared to be simply a continuation of the sharp decline seen in the preceding years.

Finally, there is internal inconsistency in the report on the strength and interpretation of the data. Specifically, although the discussion appropriately states that a variety of factors in Ethiopia "make it impossible to draw firm conclusions yet regarding the causal relationship between the observed malaria declines and LLIN and ACT scale-up," the abstract's conclusion was that: "Initial evidence indicated that the combination of mass distribution of LLIN to all children < 5 years or all households and nationwide distribution of ACT in the public sector was associated with substantial declines of in-patient malaria cases and deaths in Rwanda and Ethiopia." Readers who only saw the abstract could easily conclude incorrectly that the evidence showed that scale-up led to a reduction of the burden in Ethiopia.

## Conclusion

It is exciting that interventions to control malaria are being scaled-up across Africa and that the coming years will likely be rich with evaluations demonstrating reductions in malaria's terrible burden. Evaluations of malaria burden reduction using facility-based data could be very helpful--especially when used in conjunction with representative community-based surveys of intervention coverage, all-cause child mortality, and biomarkers, such as parasite prevalence and anaemia. However, facility-based data need to be collected, analysed, and interpreted with care, transparency, and a full recognition of their limitations. Many of these issues are described in greater detail in a guidance document from the Monitoring and Evaluation Reference Group of the Roll Back Malaria Partnership [6], and an inter-agency task force is currently exploring these issues in depth with the goal of providing practical recommendations (personal communication from S. Yoon, CDC, March 10, 2009).

## Abbreviations

LLIN: long-lasting insecticidal nets; ACT: artemisinin-based combination therapy;

## Competing interests

The authors declare that they have no competing interests.

## Authors' contributions

AKR wrote the initial draft of the manuscript, and all co-authors made substantial contributions to the content. All co-authors approved the final version.

## Funding

None.
